# Implantable wireless powered light emitting diode (LED) for near-infrared photoimmunotherapy: device development and experimental assessment *in vitro* and *in vivo*

**DOI:** 10.18632/oncotarget.25068

**Published:** 2018-04-13

**Authors:** Kohei Nakajima, Toshihiro Kimura, Hideo Takakura, Yasuo Yoshikawa, Atsushi Kameda, Takayuki Shindo, Kazuhide Sato, Hisataka Kobayashi, Mikako Ogawa

**Affiliations:** ^1^ Laboratory of Bioanalysis and Molecular Imaging, Graduate School of Pharmaceutical Sciences, Hokkaido University, Sapporo, Hokkaido, Japan; ^2^ Savior, Inc., Yokohama, Kanagawa, Japan; ^3^ Piolax Medical Devices, Inc., Yokohama, Kanagawa, Japan; ^4^ B and Plus K.K., Ogawamachi, Saitama, Japan; ^5^ Molecular Imaging Program, Center for Cancer Research, National Cancer Institute, NIH, Bethesda, MD, USA; ^6^ Presto, Japan Science and Technology Agency, Kawaguchi, Saitama, Japan

**Keywords:** light emitting diode (LED), wireless power transfer, phototherapy, near-infrared photoimmunotherapy (NIR-PIT)

## Abstract

**Purpose:**

The aim of this study was to develop and assess a novel implantable, wireless-powered, light-emitting diode (LED) for near-infrared photoimmunotherapy (NIR-PIT). NIR-PIT is a recently developed cancer therapy that uses NIR light and antibody-photosensitizer conjugates and is able to induce cancer-specific cell death. Due to limited light penetration depth it is currently unable to treat tumors in deep tissues. Use of implanted LED might potentially overcome this limitation.

**Results:**

The wireless LED system was able to emit NIR light up to a distance of 20 cm from the transmitter coil by using low magnetic fields as compliant with limits for use in humans. Results indicated that the LED system was able to kill tumor cells *in vitro* and to suppress tumor growth in implanted tumor-bearing mice.

**Conclusions:**

Results indicated that the proposed implantable wireless LED system was able to suppress tumor growth *in vivo*. These results are encouraging as wireless LED systems such as the one here developed might be a possible solution to treat tumors in deep regions in humans. Further research in this area would be important.

**Materials and Methods:**

An implantable LED system was developed. It consisted of a LED capsule including two LED sources and a receiver coil coupled with an external coil and power source. Wireless power transmission was guaranteed by using electromagnetic induction. The system was tested *in vitro* by using EGFR-expressing cells and HER2-expressing cells. The system was also tested *in vivo* in tumor-bearing mice.

## INTRODUCTION

Near-infrared photoimmunotherapy (NIR-PIT) is a new type of cancer photo-therapy based on NIR light and antibody-photosensitizer conjugates [1]. When the conjugates bind to target cells’ membranes and are exposed to NIR light, cancer specific cell death is induced without side effects, leaving adjacent normal cells undamaged [2]. A clinical trial on patients with inoperable head and neck cancer, who cannot be satisfactorily treated with chemotherapy or radiation therapy was approved by U.S. Food and Drug Administration in 2015 (https://clinicaltrials.gov/ct2/show/NCT02422979). The trial went through phase I/II successfully, suggesting that NIR-PIT may be a promising cancer treatment.

However, the procedure is not exempt from limitations. It is difficult to treat deep regions because of light absorption by tissues [3, 4]. In principle, to treat deep lesions NIR light could be delivered by using thin fiber diffusers almost anywhere in the body, e.g. via endoscopes, catheters, or needles [5, 6]. However, this procedure is a little invasive, especially considering the need for repeated treatments. In fact, repeated treatments or even repeated light exposure on a single dosing of antibody-photoabsorber conjugates can yield better therapeutic effects than a single treatment or a single light exposure [7, 8]. To try to limit the invasive character of repeated treatments in deep tissues, at the same time preserving efficacy, we investigated the possibility of using implanted NIR light emitting diode (LED) sources. Implanted LEDs are promising as they can be stable light sources for internal or interstitial NIR light exposure, thus enabling repeated light exposure of cancer lesions in deep tissues with minimally invasive procedures.

In this study, we developed a small implantable wireless LED for NIR light exposure and we have tested it *in vitro* as well as *in vivo* in tumor bearing mice. To avoid potentially harmful implantation of batteries, power supply to the implanted LED was guaranteed by electromagnetic induction from an external transmitter coil to an implanted received coil coupled with the LED. The electromagnetic field was set to be compliant with standard limits for use in humans, i.e. lower than 0.1 mT.

## RESULTS

### Wireless NIR-LED

**Figure 1:**
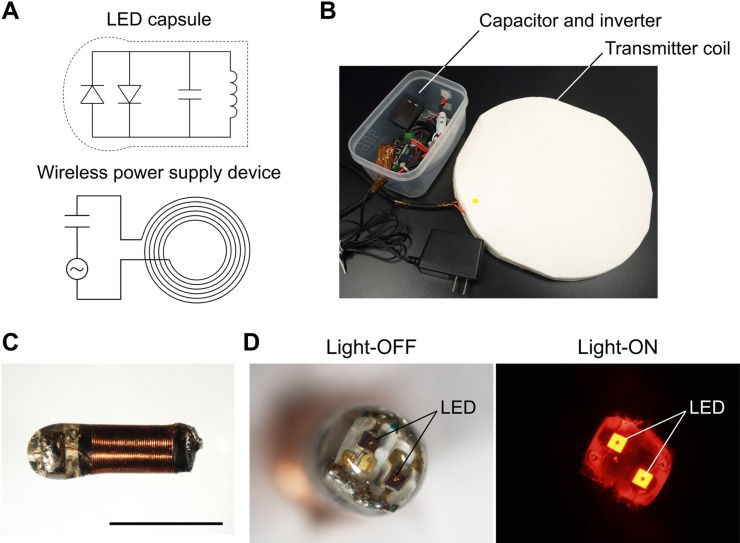
Implantable NIR-LED system (**A**) Circuit diagram of the wireless power supply device and the LED capsule. (**B**) Photograph of the wireless power supply device. The inner and outer diameters of the transmitter coil were 150 mm and 300 mm, respectively. (**C**) LED capsule. The length is about 7 mm. A copper wire was winded around a cylindrical ferrite by approximately 30 turns. The LED capsule was coated with biocompatible transparent epoxy resin. Scale bar: 5 mm. (**D**) Two LEDs at the head of the capsule in light-ON (680-700 nm) and light-OFF state.

As shown in Figure 1, we have developed the wireless NIR-LED system by coupling an external transmitter coil with the LED capsule, which included two LED sources and a receiver coil. Energy loss between the coils was minimized by using a cupper litz wire to limit the skin effect [9, 10]. Inductance and quality factor were maximized by using high magnetic permeability ferrite in the receiver coil [10]. Power transmission was further improved by limiting the error value of the resonant frequency below 0.5%. The transmitter coil was able to provide power to a receiver at a distance of up to 20 cm in low power (∼7 V, ∼0.6 A).

### *In vitro* NIR-PIT for EGFR-expressing cells

**Figure 2:**
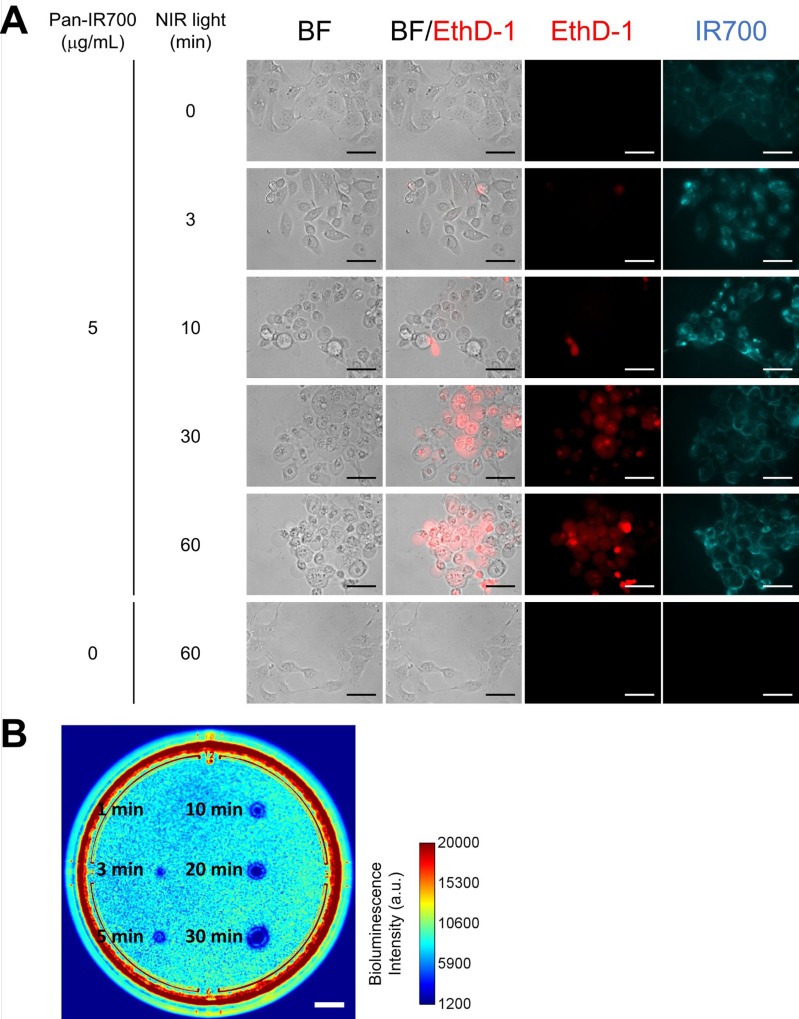
*In vitro* NIR-PIT for A431-GFP-luc cells (**A**) Phase and fluorescent microscopy of NIR-PIT treated A431-GFP-luc cells, which were pre-incubated with Pan-IR700 (5 μg/mL) at 37° C for 1 h. NIR-PIT induced cell death with cell swelling and bleb formation. EthD-1 staining showed cell death in a light dose-dependent manner. Scale bar: 50 μm (original magnification, 40×). (**B**) Bioluminescence imaging of a 10 cm dish demonstrated that luciferase activity in A431-GFP-luc cells decreased with increasing light dose. Scale bar: 1 cm.

Figure 2A shows the effect of NIR-LED irradiation on EGFR-expressing cells *in vitro*. The cytotoxic effect of NIR-PIT with the wireless LED was evaluated by using EGFR-expressing A431-GFP-luc cells. Severe cell damage such as cell swelling and bleb formation were induced by the combination of Pan-IR700 incubation and NIR light exposure (Figure 2A). Dead cell staining by using EthD-1 demonstrated that the uptake was increased in a light dose-dependent manner. No changes were observed with Pan-IR700 alone or with NIR light irradiation alone.

Decreased bioluminescence from NIR-PIT-treated cells indicated early therapeutic effects of NIR-PIT *in vitro* [11]. As shown in Figure 2B, bioluminescence imaging (BLI) demonstrated decreased luciferase activity in a light dose-dependent manner. The area with decreased luciferase activity increased with increasing light dose. These results suggested that the wireless LED induced NIR-PIT on A431-GFP-luc cells. The observed characteristics of cell death were the same as those reported in previous studies [1, 12].

### *In vitro* NIR-PIT for HER2-expressing cells

**Figure 3:**
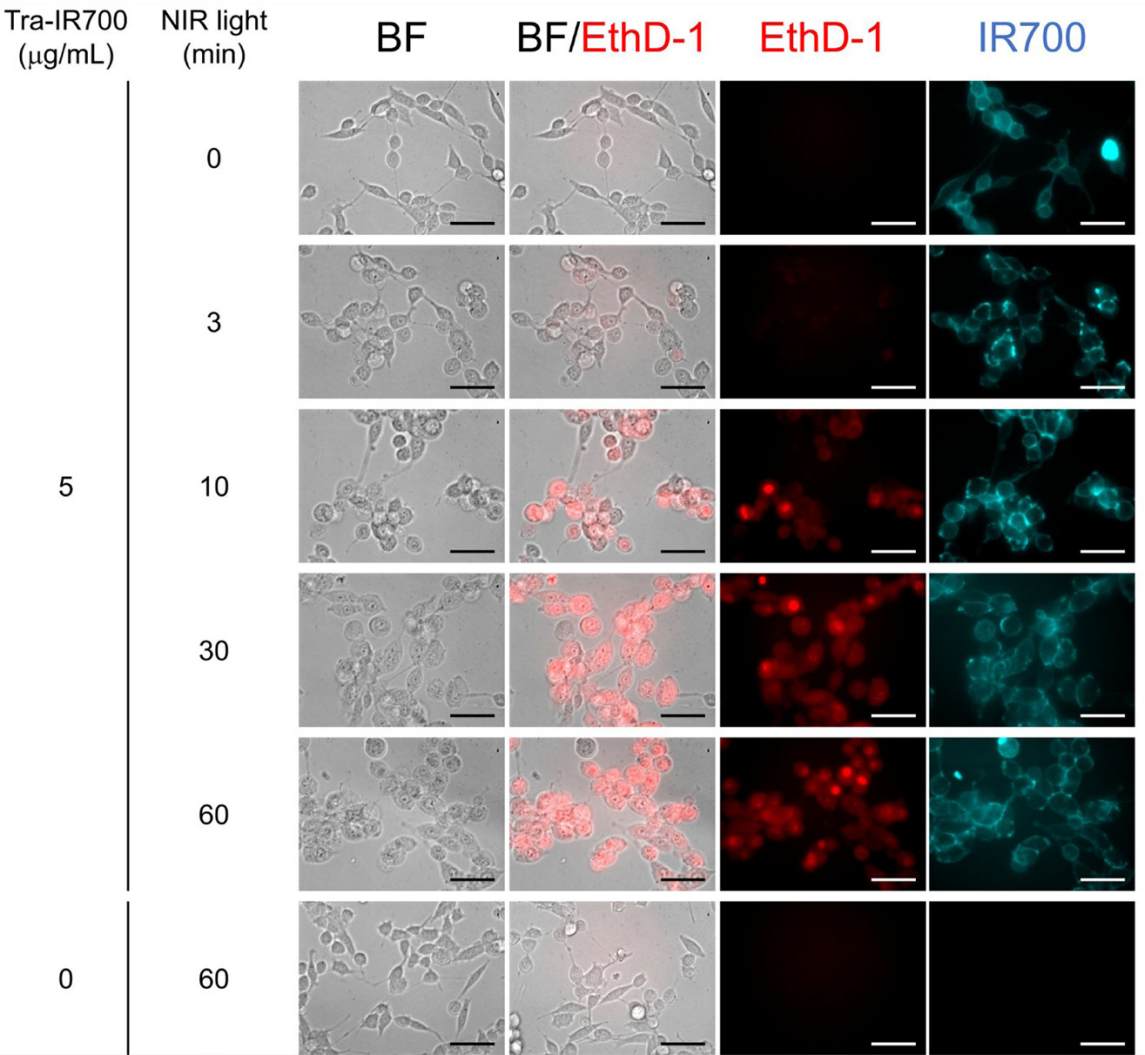
*In vitro* NIR-PIT for 3T3-HER2 cells Phase and fluorescent microscopy of NIR-PIT treated 3T3-HER2 cells, which were pre-incubated with Tra-IR700 at 37° C for 1 h. NIR-PIT induced cell swelling and bleb formation. The severity of cell damage and the uptake of EthD-1 increased with increasing light dose. Scale bar: 50 μm (original magnification, 40×).

Figure 3 shows the effect of NIR-LED irradiation on HER2-expressing cells *in vitro*. Cell swelling and bleb formation were observed in NIR-PIT (+) cells, and the severity of cell damage was dependent on the light, i.e. increased damage was observed with increasing light-dose. Fluorescence imaging showed that EthD-1 uptake increased with increasing light-dose, and that there was no cytotoxicity associated with Tra-IR700 alone nor with exposure to NIR light.

### *In vivo* NIR-PIT

**Figure 4:**
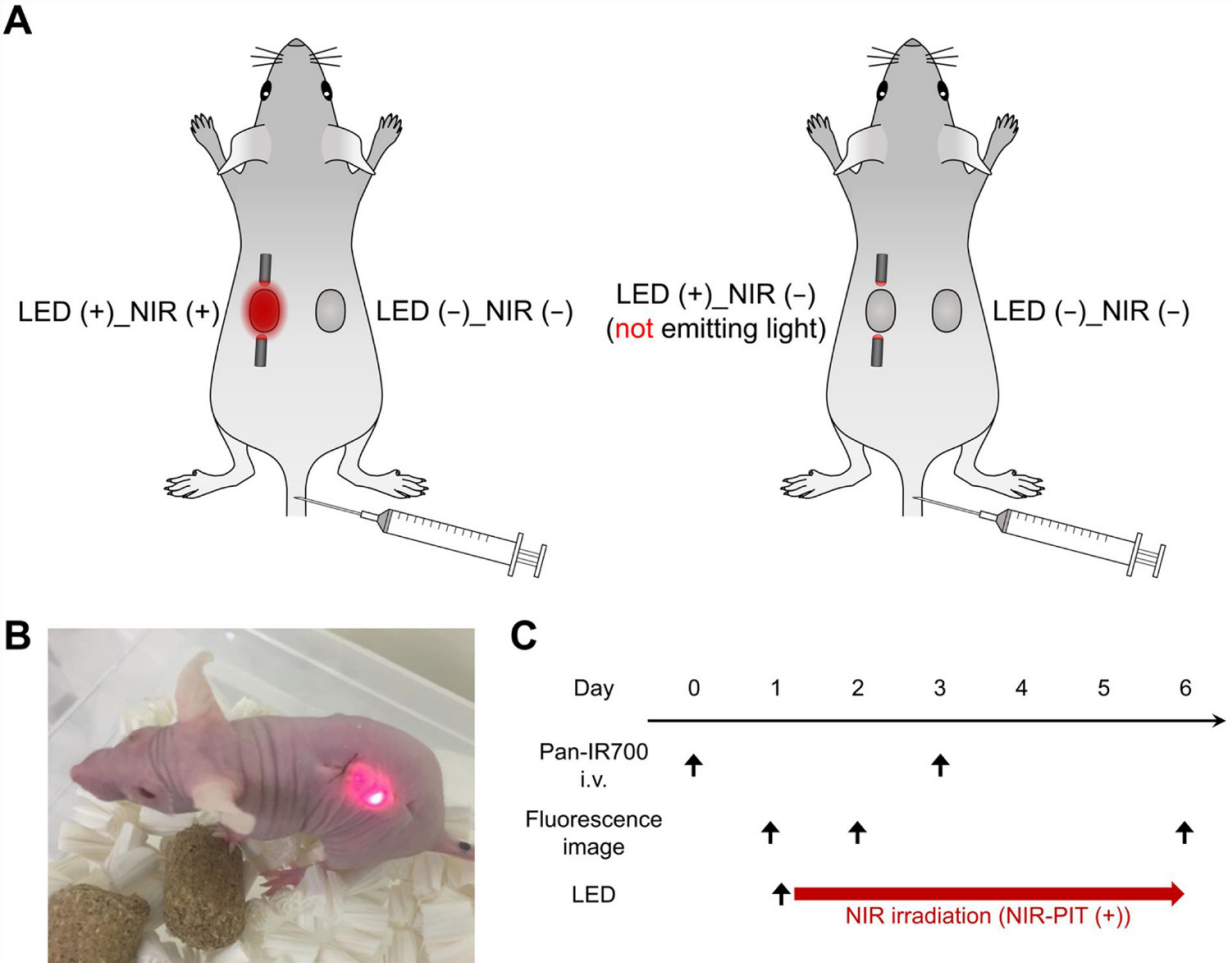
*In vivo* NIR-PIT in mice: setup (**A**) Schematic of tumor-bearing LEDs implanted mice. Treated mice (left): NIR-LED (LED (+)_NIR (+)) was implanted in one tumor and no LED in the other tumor (LED (−)_NIR (−)). Non-treated mice (right): dummy LED (LED (+)_NIR (−)) was implanted in a tumor and no LED in the other tumor (LED (−)_NIR (−)). (**B**) Photograph of a treated mouse implanted with the wireless LED. (**C**) Treatment and imaging schedule.

**Figure 5:**
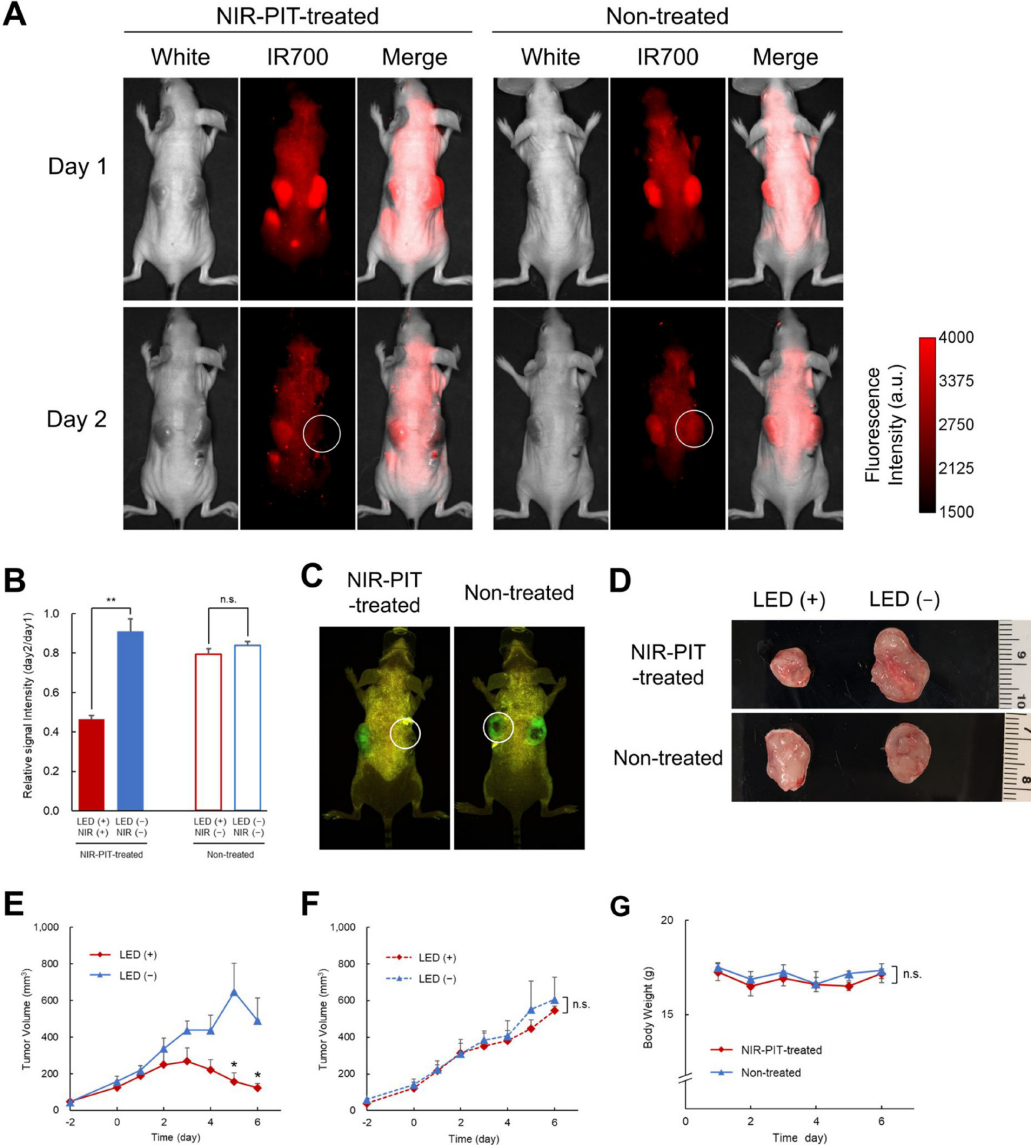
*In vivo* NIR-PIT in mice: results (**A**) IR700 fluorescence images on day 1 (top) and day 2 (bottom) of treated (left) and non-treated (right) mice. White circles indicate the NIR-LED in treated mice and the dummy LED in non-treated mice. (**B**) The relative signal intensity (day2/day1) of IR700 on LED (+)_NIR (+) tumor in treated mice was significantly lower than in tumors with no LED whereas no significant difference in relative signal intensity between the dummy LED (LED (+)_NIR (−)) tumor and the tumor with no LED was detected in non-treated mice (*n* = 6 treated mice, *n* = 3 non-treated mice) (^**^p < 0.01). (**C**) GFP fluorescence imaging on day 6 showed that the intensity in LED (+)_NIR (+) tumors in treated mice was lower than in (LED (+)_NIR (−)) tumors in non-treated mice. (**D**) *Ex vivo* images of tumors in treated (top) and non-treated (bottom) mice, showing reduced tumor size in NIR-LED exposed tumors compared to dummy LED and to tumors with no LED. (E) Tumor volume as a function of time in LED (+)_NIR (+) tumors and LED (−)_NIR (−) tumors in treated mice. The chart shows that tumor growth was suppressed in LED (+)_NIR (+) tumors (*n* = 6 treated mice). The differences between LED (+)_NIR (+) and LED (−)_NIR (−) tumors were statistically significant on day 5 and 6 (^*^p = 0.011, *p* = 0.010, respectively). (**F**) Tumor volume as a function of time in LED (+)_NIR (−) tumors and LED (−)_NIR (−) tumors in non-treated mice. No significant difference in volume was observed between the two tumors in non-treated mice (*n* = 3 non-treated mice). (**G**) Body weight as a function of time in treated and non-treated mice. The chart shows that body weights did not change during experiments in neither group (*n* = 6 treated mice, *n* = 3 non-treated mice).

To assess the effects of NIR-LED exposure *in vivo,* we conducted experiments on tumor-bearing mice injected with A431-GFP-luc cells on both sides of the dorsum, as shown in Figure 4. Figure 5 shows the results observed in treated and non-treated mice. On day 1, Pan-IR700 accumulated in both tumors (Figure 5A). On day 2, fluorescence signals of IR700 on LED (+)_NIR (+) tumor was significantly decreased in NIR-PIT-treated mice compared to non-treated (Figure 5A, 5B). On the other hand, there was no difference in relative signal intensity between LED (+)_NIR (−) tumor and LED (−)_NIR (−) tumor in non-treated mice. These results suggested that LED (+)_NIR (+) tumors were exposed to NIR light effectively [12]. On day 6, GFP fluorescence imaging demonstrated decreased signals of GFP from LED (+)_NIR (+) tumor (Figure 5C), suggesting therapeutic effects of NIR-PIT [13, 14]. The LED (+)_NIR (+) tumors in treated mice were smaller in size than LED (−)_NIR (−) tumors (Figure 5D). Moreover, tumor growth was significantly suppressed in LED (+)_NIR (+) tumors compared to LED (−)_NIR (−) tumors in treated mice (day 4, 5, and 6: *p* = 0.066, 0.011, 0.010, respectively) (Figure 5E). No significant difference was observed between treated and non-treated mice in LED (−)_NIR (−) tumors (Figure 5F). The body weights of the mice were not changed during the experiments, indicating minimal systemic toxicity due to NIR-PIT and the implanted wireless LEDs (Figure 5G).

Overall, these results suggested that the developed wireless LED was able to provide NIR-PIT *in vivo* and reduce tumors in mice.

### Histological analysis

**Figure 6:**
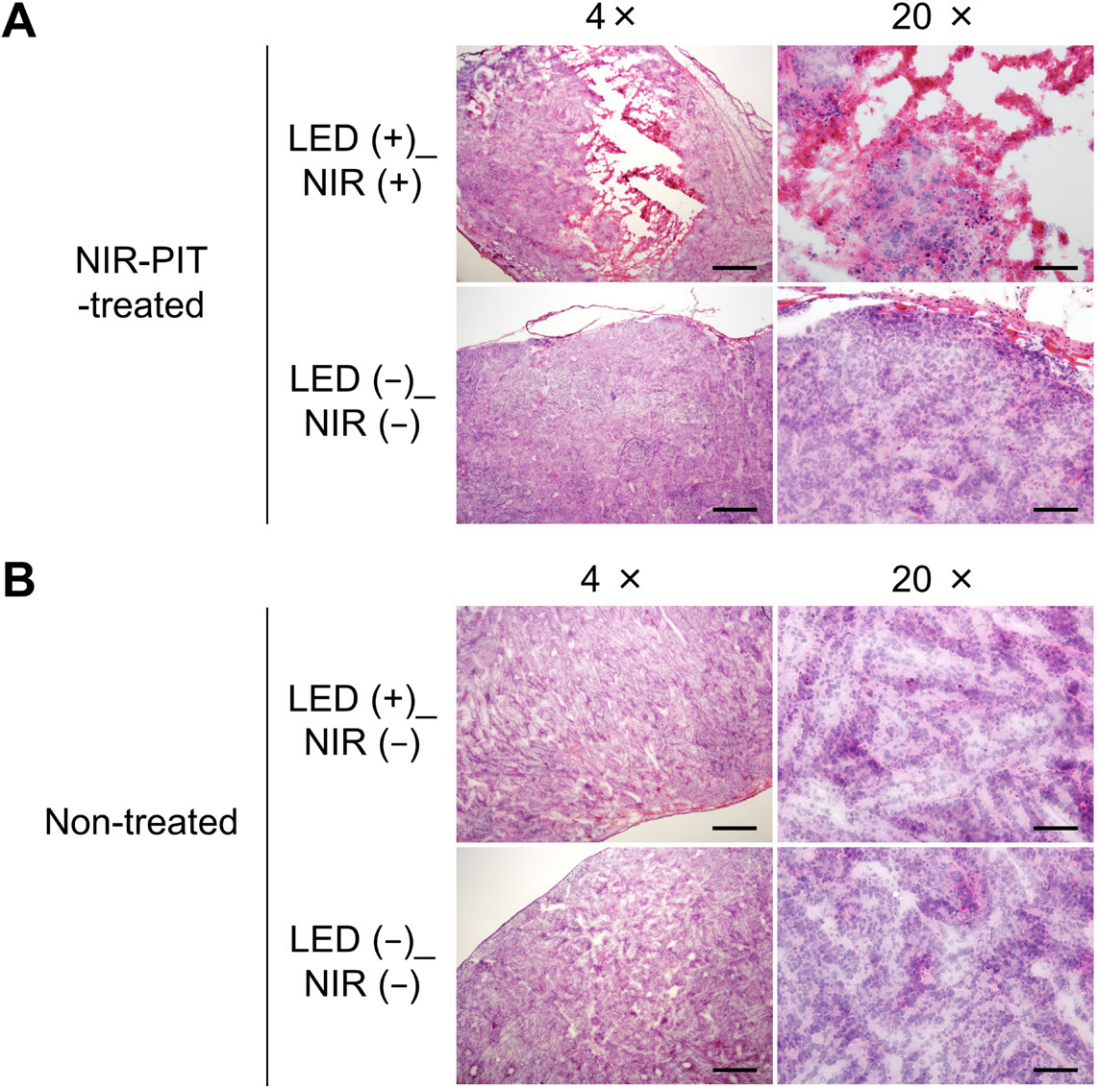
Histological analysis of NIR-PIT effects (**A**) Treated mice: tumor cells were significantly damaged in the LED (+)_NIR (+) tumor (top), whereas no apparent damage was observed in LED (−)_NIR (−) tumors (bottom). (**B**) Non-treated mice: no apparent cell damage was observed, neither in LED (+)_NIR (−) (top) nor in LED (−)_NIR (−) tumors (bottom). Scale bar: 500 μm in 4× images. Scale bar: 100 μm in 20× images.

Figure 6 shows histological analysis in treated and non-treated mice. H&E staining of LED (+)_NIR (+) tumors in treated mice demonstrated significant cell death (Figure 6A) whereas no apparent damage was observed in the other tumors, neither LED (+)_NIR (−) tumors in non-treated mice nor in LED (−)_NIR (−) tumors in treated and non-treated mice (Figure 6B).

## DISCUSSION

In this study, we have developed a prototype of implantable, wireless powered LED system for NIR-PIT. We used wireless power transmission through electromagnetic induction for supplying power from outside of the body to a receiver coil in the LED capsule, so to avoid implantation of batteries. Although the higher the electromagnetic field, the stronger the LED emission, the electromagnetic field in our system was limited to comply with the International Commission on Non-Ionizing Radiation Protection (ICNIRP) guideline [15] so to avoid possible adverse effects on the human body [16]. To limit possible immune response, the LED capsules were fully coated with biocompatible epoxy resin [17]. Interestingly, our wireless LED system could emit NIR light up to a distance of 20 cm from the power supply. This means that, ideally, most lesions in the human body can be potentially reached by irradiating from around the body. Wireless powered LED systems such as the one here proposed might potentially become devices for use in humans. Future studies on safety, efficacy, compatibility, and optimal clinical use are necessary.

Another important aspect for optimal development of implanted NIR-LED systems is related to reaching a trade-off between the light dose, which is desired to be high for increased treatment benefit, and the electromagnetic field, which needs to be low to avoid harmful effects on the human body. As a result, LED emission is limited due to limited electromagnetic field. Moreover, LED emission is also dependent on the distance between the LED/receiver coil and external transmitter coil and, also, between the small size of the LED capsule and receiver coil. Thus, especially in view of future applications in humans and in clinical settings, maximization of the LED emission is a key design issue. To try to partially tackle this issue, we reduced the error of resonant frequency between the transmitter and receiver coils and we improved the quality factor by using ferrite which has high magnetic permeability. The resulting LED emission was sufficient for treating tumors in mice.

The cytotoxic effects of NIR-PIT does not relate only to the power density (mW/cm^2^) of the light source, but rather depends on the light dose in the tissue (J/cm^2^) [11, 18]. It was reported that light doses in the range 50-150 J/cm^2^ can be effective for NIR-PIT [11, 19, 20]. In our *in vivo* experiments we had a maximum light dose of 320 J/cm^2^ which is above the minimal range and, as such, enabled tumor treatment. Another advantage of the proposed LED system compared to other solutions for NIR-PIT in deep tissues is related to the fact that the LED system is small and, once implanted, can irradiate tumors over a long period. On the other hand, conventional implantable light-delivering devices such as catheters cannot be left in the body for a long period and require recurrent implantation.

It was proven that the therapeutic benefits of NIR-PIT can be enhanced by repeated drug administration and light exposure [7]. The circulating mAb-IR700 penetrates tumors more deeply following the first NIR light exposure, thus enabling more effective treatment [21, 22]. In our *in vivo* experiments tumors were exposed to NIR light continuously following implantation. During the treatment period, it is likely that the circulating mAb-IR700 from the first and subsequent injections accumulated in the tumor and contributed to more effective treatment. Use of NIR-PIT with wireless powered implanted LED might potentially be particularly useful to treat cancers in deep tissues in patients that have not responded successfully to conventional treatments. For example, cholangiocarcinoma or bile duct cancers mostly grow along with the biliary ducts and obstruct them, causing liver dysfunction and jaundice that might kill patients [23, 24]. To avoid such complications, patients need to place a biliary stent in those parts of the biliary duct occluded or compressed by the tumor, in order to maintain the biliary flow. In these patients, combined implantation of the wireless NIR-LED along with the biliary stent might at the same time prevent occlusion of the bile duct and, also, treat the cancer in a minimally invasive way. Similarly, wireless powered implantable LED devices might also be combined with external or internal draining tubes and placed temporarily, or implanted following surgery to kill residual cancer cells.

It is clear that further research is needed to fully understand the viability of the proposed device for use in humans as well as to investigate possible issues (e.g., safety, efficacy, compatibility, and optimal criteria for clinical use). Additionally, orthotopic tumor models growing in respective organs are surely superior to the subcutaneously xenografted tumor model used in this study for evaluating clinical relevance of this LED system [25–27]. However, the results here shown are promising as the effectiveness of the proposed implantable LED for NIR-PIT was demonstrated both in *in vitro* and *in vivo*.

## MATERIALS AND METHODS

### Wireless NIR-LED

Figure 1 shows the components of the proposed wireless NIR-LED: the circuits of LED capsule and power supply device (Figure 1A); the power supply device (Figure 1B); the LED capsule (Figure 1C); and the two LED in the capsule in light-ON and light-OFF state (Figure 1D). The transmitter coil was made by winding a copper litz wire by six turns. The inner diameter was 150 mm and the outer diameter was 300 mm. The transmitter coil was connected to a capacitor (resonance frequency 1 MHz) and an inverter in series. The inverter applied high-frequency current to the coil and capacitor to generate a magnetic field.

The LED capsule was about 7 mm in length and was composed of a receiver coil, a capacitor and two LEDs as shown in Figure 1A and 1C. The two LEDs (SMT690D; USHIO OPTO SEMICONDUCTORS INC., Kyoto, Japan) emitted light at 680–700 nm. The receiver coil was made by winding a copper wire around a cylindrical ferrite (diameter: 2 mm; length: 5 mm) by approximately 30 turns. Coupling of transmitter coil and received coil provided power to the LED with electromagnetic induction. In fact, changes in the magnetic field generated by the transmitter coil induced current flow in the receiver coil thus providing electric power to the LEDs. To limit possible immune responses, the LED capsule was fully coated with biocompatible epoxy resin.

### Reagents

Panitumumab (Vectibix^®^), a fully humanized IgG2 monoclonal antibody (mAb) directed against the epidermal growth factor receptor (EGFR), was purchased from Takeda Pharmaceutical Co. Ltd. (Osaka, Japan). Trastuzumab (Herceptin^®^), 95% humanized IgG1 mAb directed against the human EGFR type-2 (HER2), was purchased from Chugai Pharmaceutical Co., Ltd. (Tokyo, Japan). Water-soluble silicon-phthalocyanine derivative, IRDye700DX NHS ester (IR700) was obtained from Li-COR Bioscience (Lincon, NE, USA). All the other chemicals used were of reagent grade.

### Synthesis of IR700 conjugated antibodies

Panitumumab or Trastuzumab (1 mg, 6.8 nmol) was incubated with IR700 (66.8 μg, 34.2 nmol) in 0.1 M Na_2_HPO_4_ (pH 8.5) at room temperature for 2 h. The mixture was purified with a gel-filtration column (Sephadex G50, PD-10; GE Healthcare, Milwaukee, WI, USA), and derived Panitumumab-IR700 (Pan-IR700) or Trastuzumab-IR700 (Tra-IR700). Protein concentration was determined with Modified Lowry Protein Assay Kit (Thermo Fisher Scientific Inc, Rockford, IL, USA) by measuring the absorption at 750 nm with a microplate reader (Infinite M200; Tecan Austria GmbH, Grödig, Austria). The concentration of IR700 was measured by absorption at 689 nm with a spectrophotometer (UV-1800; Shimadzu Corp., Kyoto, Japan) to confirm the number of fluorophore molecules to each mAb. The number of IR700 per mAb was approximately equal to three.

### Cell culture

Green fluorescence protein (GFP), luciferase-expressing A431 (A431-GFP-luc) cells, and HER2-gene-transfected NIH3T3 (3T3-HER2) cells were prepared by Dr. Kobayashi's laboratory. Cell lines were grown in RPMI-1640 (SIGMA, Saint Louis, MO, USA) containing 10% fetal bovine serum (Gibco Life Technologies, Grand Island, NY, USA) and 1% penicillin/streptomycin (Nacalai Tesque Inc., Kyoto, Japan) in tissue culture dishes in a humidified incubator at 37° C in an atmosphere composed of air (95%) and carbon dioxide (5%).

### *In vitro* NIR-PIT

A431-GFP-luc cells or 3T3-HER2 cells were seeded on 35 mm culture dishes and incubated for 24 h. After washing with phosphate buffered saline (PBS), phenol-red-free RPMI-1640 supplemented with 15 mM HEPES was added. Pan-IR700 or Tra-IR700 was then added to the culture medium at 5 μg/mL and incubated for 1 h at 37° C. Ethidium homodimer-1 (EthD-1; Molecular Probes, Eugene, OR, USA) was used to detect dead cells. The cells were irradiated from the bottom of the dish with NIR light using the wireless LED for 0, 3, 10, 30, and 60 min (0, 0.23, 0.78, 2.2, and 4.7 J/cm^2^). Power density was measured with an optical power meter (PM121D; Thorlabs, Newton, NJ, USA, 1.3 mW/cm^2^). The cells were observed by fluorescence microscope (CKX41; Olympus Corporation, Tokyo, Japan) 2 h after the onset of light exposure. To detect EthD-1, a combination of 480–550 nm excitation filter and 590 nm long pass emission filter was used. To detect IR700, a combination of 673–748 nm excitation filter and 765–855 nm band pass emission filter was used.

For BLI, after irradiation from the bottom with NIR light using the wireless LED for 1, 3, 5, 10, 20, and 30 min (0.08, 0.23, 0.39, 0.78, 1.5, and 2.2 J/cm^2^), phenol-red-free RPMI-1640 containing 150 μg/mL D-luciferin was administered to A431-GFP-luc cells. It was analyzed by using a luminescent imaging analyzer LAS-4000 mini (FujiFilm Life Sciences, Tokyo, Japan). Image analysis was conducted with ImageJ software (http://rsb.info.nih.gov/ij/).

### Animal and tumor model

The experimental protocols were approved by the Hokkaido University Animal Care Committee in accordance with the guidelines for the care and use of laboratory animals. Balb/c Slc-nu/nu nude mice (6-weeks-old, females) were purchased from Japan SLC, Inc. (Shizuoka, Japan). During procedures, mice were anesthetized with isoflurane.

Five million A431-GFP-luc cells in PBS were injected subcutaneously in right and left sides of the dorsum, and the experiments were conducted 6 days following cell injection. The greatest longitudinal diameter (length) and the greatest transversal diameter (width) of tumors were measured by using a caliper. Tumor volume was calculated following [28]: volume = length × width^2^ × 0.5. Body weight was also measured. IR700 fluorescence was evaluated using the FluorVivo Imaging System (Indec BioSystem, Santa Clara, CA, USA) (excitation: 600–640 nm, emission: 665 nm long pass). The ClearView Imaging System (Indec BioSystem, Santa Clara, CA, USA) was used to obtain fluorescence images of GFP (excitation: 452–487 nm, emission: 500 nm long pass). The region of interest was manually determined on each tumor area. Fluorescence image analysis was conducted with ImageJ software.

### *In vivo* NIR-PIT

Mice were given intravenous injection of 100 μg of Pan-IR700 on day 0 and day 3. The mice had two tumors on the dorsum and were randomized into two groups: (1) treated mice: a tumor with NIR-LED (LED (+)_NIR (+)) and a tumor without LED (LED (−)_NIR (−)); (2) non-treated mice: a tumor with implanted dummy LED (not emitting NIR light) (LED (+)_NIR (−)) and a tumor without LED (LED (−)_NIR (−)) (Figure 4A). The dummy LEDs were used to investigate possible tumor growth inhibition effects due to damage of blood vessels during LED implantation. LED capsules were implanted subcutaneously in a way that the tumor was in between the two LED capsules (Figure 4B). Mice were bred on the wireless power supply device after LED implantation. Figure 4C shows the treatment and imaging schedule. NIR-LED irradiation of LED (+)_NIR (+) tumor in treated mice was continuous until day 6. IR700 fluorescence imaging was assessed on day 1 and day 2. GFP fluorescence imaging was assessed on day 6.

### Histological analysis

To evaluate the histological changes, mice were euthanized on day 6. Extracted tumors were frozen in optimal cutting temperature compound, and 10 μm frozen slice sections were prepared. Hematoxylin and eosin (H&E) staining was performed according to standard protocols. The slides were imaged with an Olympus BX41 microscope (Olympus Corporation, Tokyo, Japan) with 4× and 20× objective lenses.

### Statistical analysis

The measured quantities were expressed as means ± SEM (standard error of the mean). Statistical analyses were carried out using JMP Pro 12.2.0 software (SAS Institute Inc., Cary, NC, USA). Possible differences in mean values were assessed by using student's *t* tests. Values of *p* < 0.05 were considered statistically significant.

## Abbreviations

BLI: bioluminescence imaging
EGFR: epidermal growth factor receptor
EthD-1: ethidium homodimer-1
HER2: human epidermal growth factor receptor type-2
ICNIRP: International Commission on Non-Ionizing Radiation Protection
IR700: IRDye700DX
LED: light emitting diode
mAb: monoclonal antibody
NIR-PIT: near-infrared photoimmunotherapy
